# Protein complexes and neighborhoods driving autophagy

**DOI:** 10.1080/15548627.2020.1847461

**Published:** 2020-11-13

**Authors:** Devanarayanan Siva Sankar, Jörn Dengjel

**Affiliations:** Department of Biology, University of Fribourg, Fribourg, Switzerland

**Keywords:** Autophagy, affinity purification, mass spectrometry, protein-protein interactions, proximity labeling, quantitative proteomics

## Abstract

Autophagy summarizes evolutionarily conserved, intracellular degradation processes targeting cytoplasmic material for lysosomal degradation. These encompass constitutive processes as well as stress responses, which are often found dysregulated in diseases. Autophagy pathways help in the clearance of damaged organelles, protein aggregates and macromolecules, mediating their recycling and maintaining cellular homeostasis. Protein-protein interaction networks contribute to autophagosome biogenesis, substrate loading, vesicular trafficking and fusion, protein translocations across membranes and degradation in lysosomes. Hypothesis-free proteomic approaches tremendously helped in the functional characterization of protein-protein interactions to uncover molecular mechanisms regulating autophagy. In this review, we elaborate on the importance of understanding protein-protein-interactions of varying affinities and on the strengths of mass spectrometry-based proteomic approaches to study these, generating new mechanistic insights into autophagy regulation. We discuss in detail affinity purification approaches and recent developments in proximity labeling coupled to mass spectrometry, which uncovered molecular principles of autophagy mechanisms.

**Abbreviations**: AMPK: AMP-activated protein kinase; AP-MS: affinity purification-mass spectrometry; APEX2: ascorbate peroxidase-2; ATG: autophagy related; BioID: proximity-dependent biotin identification; ER: endoplasmic reticulum; GFP: green fluorescent protein; iTRAQ: isobaric tag for relative and absolute quantification; MS: mass spectrometry; PCA: protein-fragment complementation assay; PL-MS: proximity labeling-mass spectrometry; PtdIns3P: phosphatidylinositol-3-phosphate; PTM: posttranslational modification; PUP-IT: pupylation-based interaction tagging; RFP: red fluorescent protein; SILAC: stable isotope labeling by amino acids in cell culture; TAP: tandem affinity purification; TMT: tandem mass tag.

## Introduction

The majority of proteins are degraded via two pathways: the ubiquitin proteasome system and autophagy. In comparison to the specific degradation of the ubiquitin proteasome system, autophagy is thought to degrade substrates nonselectively and selectively due to cargo receptors [1]. Thus, autophagy summarizes constitutive lysosomal degradation pathways and stimulus-dependent stress responses to preserve cellular homeostasis. Macromolecules, protein aggregates and organelles are degraded and recycled to accomplish the energy demands of the cell and to restock basic building blocks for anabolic processes. Autophagy acts in general in a cytoprotective manner and its dysregulation has been linked to various diseases such as neurodegeneration, cancer and metabolic syndrome [2]. Autophagy has been broadly categorized into three different subtypes: macroautophagy, microautophagy and chaperone-mediated autophagy, which differ in the mode of transport and delivery of substrates for lysosomal degradation. Microautophagy describes an invagination of endosomal or lysosomal membranes that engulf cytoplasmic substrates for degradation [3]. Chaperone-mediated autophagy is probably the most selective subtype of autophagy. Recognition and unfolding of substrates carrying KFERQ-like motifs by cytosolic chaperone HSPA8/HSC70 (heat shock protein family A [Hsp70] member 8) determines this selectivity. Unfolded proteins are translocated across lysosomal membranes for degradation by LAMP2A, a lysosomal membrane protein [4,5]. In a process termed endosomal microautophagy, substrates carrying the KFERQ motif are selectively recognized by cytosolic HSPA8 chaperones and targeted for degradation to late endosomes instead of the lysosomes via binding to phosphatidylserine on endosomal membranes [6]. Macroautophagy, hereafter referred to as autophagy, is an intracellular degradation pathway that starts with the formation of a double-membraned vesicle called autophagosome, which enwraps cytoplasm targeted for lysosomal degradation. Autophagosome biogenesis consists of distinct hierarchical phases starting with the formation of a cup-shaped membrane called phagophore, followed by elongation of the phagophore, maturation, closure, fusion with endosomes, and finally with lysosomes leading to the formation of autolysosomes [7,8]. Autophagy has been shown to play a critical role in selectively clearing damaged organelles (mitochondria, peroxisomes etc.), infectious agents and protein aggregates [2].

**Figure 1.**
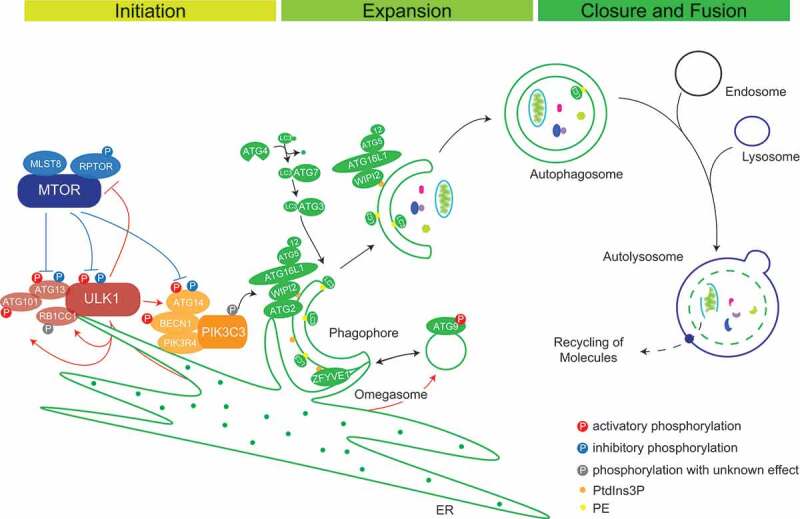
Schematic representation of autophagosome biogenesis and maturation. MTORC1 inhibits autophagy via its inhibitory phosphorylations on ULK1 and ATG13 under nutrient-rich conditions. Under stress, autophagy is activated via the formation of an active tetrameric ULK1 initiation complex to promote autophagosome nucleation. PtdIns3K complex I catalyzes the production of PtdIns3Ps, which contributes to phagophore nucleation and omegasome formation. PtdIns3P-binding proteins like ZFYVE1/DFCP1 and WIPIs decorate the omegasomes. WIPI2 interaction with ATG16L1 mediates the recruitment of the ATG12–ATG5-ATG16L1 complex for the conjugation of LC3-I to PE and phagophore expansion and maturation. Additionally, lipid sources from ATG9 vesicles, ATG2A/B recruited by WIPIs and from cell membranes collectively help in expanding the phagophore membrane. Double-membraned autophagosomes fuse with lysosomes to form autolysosomes and their content is degraded by lysosomal hydrolases

Over the last few decades, more than 40 autophagy-related (*ATG*) genes/proteins have been reported in yeast. Most of these are conserved between yeast and mammals and have crucial roles in the progress of autophagy [9]. The canonical core pathway is regulated by six conserved protein complexes [10]: (i) the ULK1 (unc-51 like autophagy activating kinase 1)-ULK2 complex, which is critical for autophagy initiation; (ii) the ATG9 system, which provides membranes for autophagosome generation; (iii) the class III phosphatidylinositol 3-kinase complex (PtdIns3K), which phosphorylates the lipid phosphatidylinositol (PtdIns) generating PtdIns-3-phosphate (PtdIns3P) that serves as binding site for protein recruitment; (iv) the ATG2-WIPI complex, which is important for membrane expansion, (v) the ATG12 and (vi) Atg8-family proteins ubiquitin-like conjugation systems. The latter being important for phagophore expansion and cargo recruitment [11]. We will discuss the roles of these complexes in more detail in the following paragraphs (Figure 1).

### Protein complexes regulating autophagy

Initiation of autophagy is executed by two kinase complexes, the ULK1 complex, the mammalian homolog of yeast Atg1 complex, and secondly, the PtdIns3K complex [12]. ULK1 (or its homolog ULK2), a Ser/Thr kinase, phosphorylates itself, and its complex members ATG13, ATG101 and RB1CC1/FIP200. Together, they form the active tetrameric autophagy initiation complex, e.g., in response to starvation [13,14]. Endoplasmic reticulum (ER) recruitment of this complex happens through direct interaction of ULK1 and RB1CC1 via their FFAT motifs with ER membrane proteins VAPA (VAMP associated protein A)-VAPB, forming active phagophore initiation sites on the ER membrane. Additionally, VAPs interact with WD repeat-containing protein WIPI2; a membrane-associated protein forming a tethering complex and strengthening ER-phagophore contact, making ER an essential organelle for autophagosome biogenesis [15]. ATG9, a multi-membrane spanning protein, predominantly localizes at the trans-Golgi network and endosomes. It is postulated to organize a lipid source for phagophore generation and expansion due to its colocalization to the phagophore membrane [16]. ATG9-containing vesicles fuse with ATG16L1-containing vesicles to progressively promote autophagosome biogenesis [17] (Figure 1).

There are two distinct PtdIns3K complexes. Complex I promotes autophagy and consists of the lipid kinase PIK3C3/VPS34, the adaptor protein PIK3R4/VPS15, ATG14, which helps in ER membrane tethering, the stabilizing subunit BECN1/Beclin-1, and the accessory subunit NRBF2. In complex II, UVRAG replaces ATG14 and NRBF2. Complex II takes over functions in later stages of autophagy [12]. ULK1 helps recruiting the PtdIns3K complex I to active initiation sites by phosphorylating the following proteins: (a) AMBRA1 on amino acid residues Ser465 and Ser635 to release the tethered PIK3C3-BECN1 complex toward the ER from AMBRA1, which is bound to microtubules via DYNLL1 and DYNLL2 (dynein light chain LC8-type 1/2) [18], (b) PIK3C3 on Ser249 [18,19], (c) BECN1 on Ser15 (human)/Ser14 (murine) stabilizing the complex [20], and (d) ATG14 on Ser29 activating autophagy by increasing PIK3C3 activity [21]. The PtdIns3K complex increases production of PtdIns3P at phagophore formation sites, where PtdIns3P binding protein ZFYVE1/DFCP1 accumulates, leading to crescent-shaped growing phagophores called omegasomes [22]. These structures provide strong platforms for the binding of WIPI1 and WIPI2 involved in nascent autophagosome biogenesis [23]. WDR45B/WIPI3 and WDR45/WIPI4 positively affect signaling events up- and downstream of PtdIns3P production, controlling the size of autophagosomes [24]. In addition, human homologs of yeast lipid transfer protein Atg2 (ATG2A and ATG2B) decorate phagophores via their interaction with WIPIs [24]. ATG2s are involved in maintaining a membrane tether or contact site between ER and phagophores leading to expansion of the growing phagophores by direct lipid transfer [25]. Later, they help in autophagosome membrane closure [26] (Figure 1).

Ubiquitin-like modifiers (UBLs) play important roles in autophagosomal cargo recruitment and maturation. ATG12 and the family of Atg8 homologs are characterized by ubiquitin folds as UBLs. As ubiquitin, respective proteins are activated and transferred to target proteins by sets of sequential reactions [27]. ATG7, a ubiquitin E1-like enzyme, activates the C-terminal glycine of ATG12 and hands it over to ATG10, an E2-like enzyme. ATG10 transfers ATG12 to its target ATG5, which in turn binds to ATG16L1 to form the ATG12–ATG5-ATG16L1 trimeric complex [27–29]. Atg8 homologs are UBLs classified into two subfamilies: the LC3 family consisting of MAP1LC3A, LC3B, LC3B2 and LC3C and the GABARAP family consisting of GABARAP, GABARAPL1, and GABARAPL2 (hereafter collectively referred to as LC3s) [30]. LC3s are anchored in membranes by conjugation to phosphatidylethanolamine (PE). First, cleavage at the C terminus by ATG4B proteases exposes a terminal glycine residue and activates LC3 (LC3-I) to get bound by ATG7 [31]. The E2-like enzyme ATG3 and the ATG12–ATG5-ATG16L1 complex, which has E3 ligase activity, transfer LC3-I (cytosolic) to PE (LC3-II). This happens at active sites of autophagosome biogenesis due to recruitment of the ATG12–ATG5-ATG16L1 complex via the interaction between ATG16L1 and WIPI2 [29]. LC3s are predominantly involved in the selective capture of substrates for degradation. Autophagy receptors bind to LC3s via so-called LC3-interacting regions (LIRs) [32]. However, LC3s also help in the maturation and closure of nascent autophagosomes [33]. In general, autophagosomes fuse with endosomes to form amphisomes before they finally fuse with lysosomes to form autolysosomes for degradation of their contents [34]. Fusion of mature autophagosomes with lysosomes is carried out in a concerted manner by the action of RAB and SNARE proteins and membrane tethering complexes, most importantly by RAB7A, STX17, SNAP29, VAMP8 and the HOPS complex [35].

Delicate balance between anabolism and catabolism is essential for the survival of cells. Anabolism is positively regulated by the master regulator of cell growth MTORC1, a conserved Ser/Thr kinase complex consisting of the kinase MTOR (mechanistic target of rapamycin kinase), and the regulatory subunits MLST8 and RPTOR. MTORC2, composed of MTOR, RICTOR and MLST8, is thought to positively modulate cell proliferation [36]. MTORC1 is well known to inhibit catabolic pathways, including autophagy where inhibitory phosphorylations of Ser637 and Ser757 on ULK1 and Ser258 on ATG13 prevent them from forming the active autophagy initiation complex. Nutrient deprivation or rapamycin treatment inhibit MTORC1 activity, thus enabling the activation of autophagy *via* dephosphorylation of MTORC1 sites and subsequent activating phosphorylations of, for example, ATG13 by ULK1. Under starvation conditions, dephosphorylation of MTORC1 target sites on ULK1 is carried out by the heterotrimeric PP2A protein phosphatase, consisting of the catalytic subunit PPP2CA, the scaffolding subunit PPP2R1B/PRL65, and the regulatory subunit PPP2R2A/B55alpha [37]. Additionally, the energy demand activates AMP-activated protein kinase (AMPK), an AMP:ATP ratio sensor. AMPK activates the ULK1 complex by phosphorylating Ser317, Ser659, and Ser777 on ULK1 itself and Ser224 on ATG13. Active ULK1 catalyzes autophosphorylations on different sites, among others Thr180, Ser1042, and Thr1046 [14].

The dynamic organization of sequential events in autophagy is highly regulated by protein-protein interactions (PPIs) and post-translational modifications (PTMs) happening at the right spatial and temporal resolution. To study PPIs *in vitro* and *in vivo*, different techniques have been used [38]. The strength of the interactions, either strong/permanent or weak/transient, determine the efficient usage of a specific method. In order to obtain a global, unbiased picture of PPIs, mass spectrometry (MS)-based proteomic approaches studying PPIs and neighborhoods have gained much attention lately [39]. New developments allow the study of strong and weak interactions, supporting the construction of hierarchical networks. Co-immunoprecipitation methods like tandem affinity purification (TAP) enrich strong interactions. To capture weak interactions, crosslinking coupled to affinity purification (AP), proximity labeling (PL) and bio-orthogonal chemistries coupled to MS are now widely used. In the following chapters, we summarize the different MS-based methods to identify PPIs of different strengths and highlight their usage to study the regulation of autophagy.

### Protein-protein interactions and their regulation

**Figure 2.**
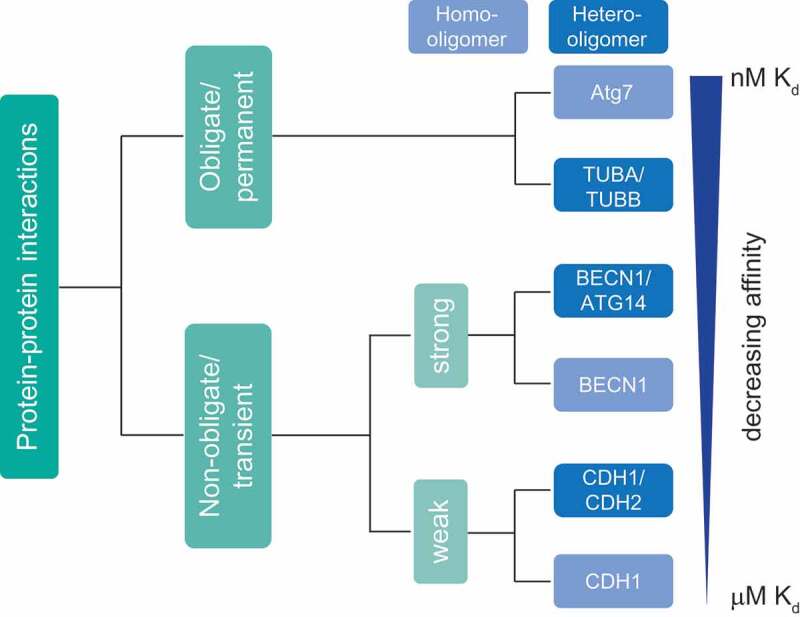
Classification of protein-protein interactions based on stability and binding affinity. Affinity is inversely proportional to the dissociation constant K_d_. Based on stability and binding affinity, PPIs can be classified into quasi-permanent/obligate and transient/non-obligate interactions. In contrast to permanent complexes, transient complexes are dynamic with proteins associating and dissociating. Transient complexes can be subclassified as strong and weak based on affinity and temporal profile of interactions. Moreover, PPIs can be classified based on composition as homo-oligomers with identical proteins interacting and hetero-oligomers with non-identical chains interacting. Often, interactions change due to physiological conditions rather reflecting a continuous than a “static” classification

Dynamic PPIs regulate virtually all biological processes, e.g., metabolism, DNA replication, protein synthesis, as well as autophagy. Interactions can range from simple binary interactions to complex multimeric interactomes and are essential to maintain efficient functioning of cells thereby balancing cell physiology. Hence, understanding these interactions is crucial for revealing molecular functions and disease-associated mechanisms in order to design potent therapeutics [40]. Based on stability, PPIs can be classified into obligate, when binding partners are not stable by themselves, and non-obligate interactions of otherwise stable protomers. Based on binding affinity, i.e. the temporal profile of interactions, PPIs can be classified into permanent and transient interactions [41]. However, often PPIs do not fall into a static classification and a continuum exists between quasi-permanent and transient interactions. As a given protein can also interact with different proteins and form several distinct complexes *in vivo*, the discrimination between obligate and non-obligate interactions may not be straight forward and depend on a given (patho)physiological condition [42,43]. In addition, permanent interactions often involve proteins that are unstable as monomers and that function in complexes. Thus, due to experimental limitations and in order to simplify a classification based on *in vivo* observations, only obligate PPIs might be regarded as quasi-permanent (Figure 2). In contrast, structurally stable proteins that interact with different proteins by undergoing association and dissociation reactions form transient/non-obligate PPIs, i.e. time-limited interactions. Based on affinity and temporal quality, transient interactions can be further classified as strong and weak transient interactions. Moreover, protein complexes are classified based on composition as homo-oligomeric (having identical protein members) and hetero-oligomeric complexes (having non-identical protein members).

Homo-oligomers generally form highly stable, permanent structures and are symmetric in nature. They also form scaffolds for stable interactions with other macromolecules. For example, yeast Atg7 is an E1-like enzyme that forms a functionally-active homo-dimeric complex (K_d_ = 1 nM). As described above, ATG7 plays a major role in the two ubiquitin-like conjugation pathways involved in autophagy: ATG12–ATG5-ATG16L1 and LC3–PE generation [44]. TUBA (tubulin alpha) and TUBB (tubulin beta) form a quasi-permanent heterodimer (K_d_ ≈ 84 nM [54–123 nM]), which polymerizes to form dynamic microtubules. These help in autophagosome formation, motility and fusion with lysosomes [43,45,46]. Another example of a quasi-permanent, homo-oligomeric complex is CASTOR1 (cytosolic arginine sensor of MTORC1), a protein that forms a stable homodimer and acts as an amino acid sensor by binding to arginine [47].

In contrast, transient complexes associate and dissociate based on physiological conditions. Proteins can function either independently or by forming a complex [42,48,49]. Transient complexes have a wide range of affinities and temporal stabilities, depending on the requirements in cells. Hetero-oligomers often form transient complexes of varying stability. Based on their characteristics, transient complexes are classified into strong and weak transient interactions. A strong transient interaction would have a higher affinity and longer lifetime compared to a weak transient interaction. For instance, ATG12 is activated by ATG3 forming a strong, transient hetero-oligomeric complex (K_d_ = 50 nM) [50]. BECN1, a protein that forms a strong transient homodimer, remains inactive in nutrient-rich conditions. However, upon a physiological stress stimulus leading to activation of autophagy, a BECN1 monomer can interact strongly with ATG14 (K_d_ = 3.2 µM) to form an active hetero-oligomeric PtdIns3K class-III complex [51]. Weak transient interactions are continuously broken and formed and difficult to capture by biochemical/analytical approaches. For example, cadherins are adhesion proteins, which mediate cell-cell communication. Expression of different cadherins in distinct cell types helps in spatial positioning of respective cells during development and maintaining their 3-D architecture, crucial for tissue function. In order to maintain specific cell-cell communication, CDH1/E-cadherin forms weak homodimers (K_d_ = 160 µM). CDH1 forms also hetero-oligomers with CDH2/N-cadherin, which interact stronger, mediating contacts of different cell types. Autophagy ensures optimal cell growth via regulating the abundance of CDH1, a protein involved in CTNNB1 (catenin beta 1)-WNT signaling [52–54] (Figure 2). SNXs (sorting nexins), a class of peripheral membrane proteins important for endosomal sorting, can oligomerize via their Bin/Amphiphysin/Rvs (BAR) domains to perform vesicle to tubular membrane remodeling. SNX4 forms weak homodimers to perform tubule formation of membranes. Under certain physiological conditions such as autophagy, SNX4 can form a hetero-oligomeric complex with SNX7 to mediate autophagosome assembly and maturation [55–57]. Overall, the above examples highlight the variety of affinities of protein-protein interactions important for autophagy.

Structural features of proteins, such as motifs and domains, modulate an array of interactions. In addition, differential interactions can be modulated by factors such as pH, compartmentalization, local concentration of ions, and covalent modifications like phosphorylation and ubiquitination. The above features collectively impart specificity to PPIs and mediate oligomerization of proteins meant to interact in a crowded environment [42,58].

### Mass spectrometry-based proteomic approaches to study protein-protein interactions

Due to the importance of PPIs, numerous cell biological and biochemical methods exist to study them *in vitro* and *in vivo*. This review cannot comprehensively summarize all approaches in detail (e.g., see [59] for more technical details), but we would like to list important, well-established examples of *in vitro* and *in vivo* methods, focusing on MS-based approaches. *In vitro* methods are techniques carried out in a controlled environment after cell lysis and allow the analysis of binary as well as oligomeric interactions. Examples are affinity purifications, size-exclusion chromatography, protein arrays, enzyme-linked immunosorbent assay, and biophysical methods such as isothermal calorimetry, microscale thermophoresis, and surface plasmon resonance. Powerful MS-based *in vitro* methods are: AP-MS, chemical cross-linking MS (XL-MS), co-fractionation coupled to MS (CoFrac-MS) and PL-MS methods. *In vivo* methods are techniques carried out in a living organism and commonly address binary interactions [60]. Examples are yeast two-hybrid analyses or image-based analyses, such as bimolecular fluorescence complementation, Förster resonance energy transfer, bioluminescence resonance energy transfer, and proximity ligation assays [61].

MS-based proteomic studies are widely used to understand mechanisms in biological pathways by analyzing PPIs. Technological advancements in terms of MS-instrumentation, liquid chromatography (LC), sample preparation methods, techniques used to enrich interactomes, high throughput analyses, and bioinformatics data interpretation have greatly increased the usage of MS-based approaches. The ultimate goal of proteomic studies is to comprehensively characterize the proteome, protein expression levels, PTMs, and build functionally relevant protein networks. Based on the nature of protein identifications and characterizations, two approaches are discriminated: top-down and bottom-up proteomics [62].

In top-down proteomics, intact proteins are ionized, followed by fragmentation and measurement in a high-resolution mass spectrometer. This approach provides a complete characterization of proteoforms including PTMs. The bottom-up proteomic approach involves chemical or enzymatic digestion of proteins into peptides prior to ionization and MS measurement. This method is easily automatable and generally helps in the identification of large numbers of proteins compared to top-down approaches, which require a more sophisticated front-end biochemistry to generate samples for MS analyses [63].

Different strategies in MS sample acquisition exist: data-dependent acquisition (DDA), data-independent acquisition (DIA), both unbiased discovery methods, and directed/targeted proteomics approaches. These strategies differ in the depth or coverage of protein identifications. DDA is based on a selection and identification of the most abundant ions in the sample. In DIA, a relatively new method, ions are not selected. Entire groups of ions are measured, generating a “digital map” of the entire sample [39,64–70]. Whereas DIA approaches lead to less missing values across experiments, data interpretation is more challenging. Targeted proteomics approaches deal with the specific isolation and measurement of predefined ions, making it more reproducible compared to discovery proteomics experiments.

The aforementioned strategies have advantages and dis-advantages, leading to trade-offs in sensitivity, specificity, reproducibility, accuracy, and dynamic range (for more information on these strategies, see [39,64–70]). In general, different tools or methods are available for every step in the experiment starting from sample isolation and preparation, MS data acquisition, data analysis, and statistical interpretation. Given the applicability of MS in a wide range of biological questions, choosing the right combination of tools is essential for answering the complex biochemical questions underlying autophagy regulation.

### Quantitative proteomics

As signal intensities recorded by MS depend on ionization properties of respective biomolecules, MS is not a truly quantitative analytical approach. Therefore, quantitative proteomics strategies have been developed that allow a systematic quantification of samples revealing changes between measured proteomes. Quantitative information is key to characterize molecular pathways both at protein and PTM level. Quantification can be performed in two ways, either absolute or relative. Absolute quantification is possible by comparing signal intensities of biomolecules with signals of known amounts of respective synthetic/purified standard substances. Relative quantification is performed by comparing ion intensities between different samples. Quantification of proteins or peptides can be performed label-free or via stable isotope labeling strategies, the latter being more accurate as samples can be analyzed in single MS experiments. Labeling approaches add tags that differ in mass but do not interfere with ionization properties. Tags can be incorporated at protein or peptide level, enzymatically, chemically, or metabolically [71].

Metabolic labeling summarizes strategies that commonly lead to the *in vivo* incorporation of ^13^C and ^15^N isotope-labeled metabolites such as amino acids. In one of the most common approaches, stable isotope labeling by amino acids in cell culture (SILAC), labeled lysine (K) and arginine (R) variants are used. Commonly three different SILAC labels are used to perform relative quantifications between three biological conditions [72,73]. SILAC is majorly used in mammalian cell culture studies. Metabolic labeling is advantageous as it allows mixing of cells prior to biochemical perturbations, thus improving quantification accuracy. Enzymatic labeling is being carried out during MS sample preparation, most commonly used in bottom-up proteomics experiments. For example, the use of ^18^O-labeled water allows the incorporation of ^18^O to neo-C-termini generated by proteolytic digestion [74,75]. Chemical methods utilize the availability of reactive N-termini and amino acid side chains to incorporate chemical groups. Isobaric mass tags are linked to reactive N-terminal and epsilon amino groups of lysine residues via N-hydroxysuccinimide (NHS) chemistry. These tags are made up of a peptide reactive group, a stable isotope-labeled reporter ion, and a balancer group. Prominent examples are isobaric tags for relative and absolute quantification (iTRAQ) and tandem mass tag (TMT) labeling. These tags have similar chemical structures, differing in positions, numbers, and combinations of ^13^C and ^15^N isotopes, and can be highly multiplexed [76,77]. Currently, up to 16 samples can be quantified in a single experiment on a routine basis [78,79]. Due to new bioinformatics approaches, label-free quantification based on peptide precursor (DDA) or fragment ion (DIA) intensities is a widely used and cheap method for robust relative quantification [68,80]. The aforementioned methods have advantages and disadvantages pertaining to the nature of the quantification method, sample type, multiplexing capacity, cost-effectiveness, quantification accuracy, sensitivity, and proteome coverage [68,81,82].

### Affinity purification-mass spectrometry to study protein-protein interactions

**Figure 3.**
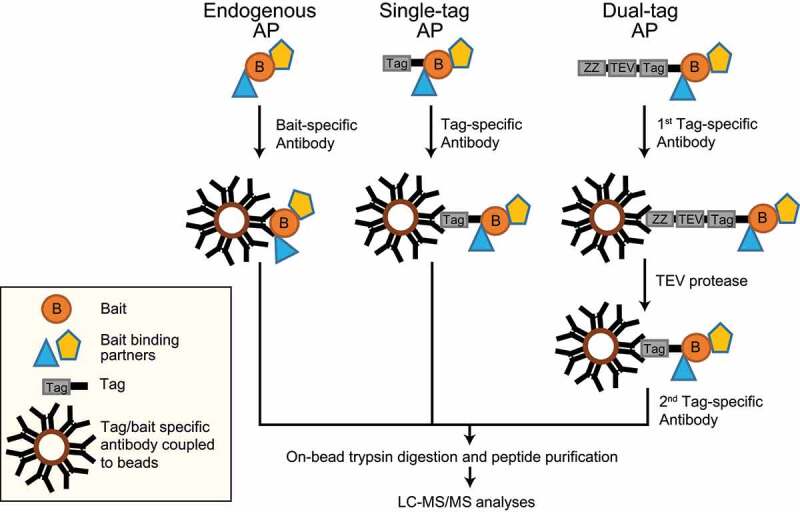
AP-MS approaches. (A) AP of endogenous proteins is a method to identify bait interactors using bait-specific antibodies, which are coupled to beads. (B) Single-tagged bait proteins are expressed in cells and affinity purified using a tag-reactive antibody. (C) The TAP-tag consists of two biochemical tags used for purification. In the classical TAP, the first tag is the ZZ domain of protein-A followed by a TEV protease cleavage site and a second tag. This method involves two consecutive steps of purification

AP-MS is one of the widely used methods to identify and characterize PPIs. The principle of this method is to isolate a protein of interest (referred to as bait protein) and to identify/quantify its interacting proteins by MS. For AP, either an antibody recognizing the endogenous protein or the expression of a tagged-protein variant in combination with a tag-recognizing structure are commonly used. Affinity tags or antibodies are captured by other macromolecules covalently attached to a solid matrix such as agarose beads. Macromolecules can be oligonucleotides (DNA/RNA), proteins, such as proteins A and G binding the F_c_ parts of antibodies, peptides and lipids [83–86]. In general, antibodies are coupled to beads and are used to capture tagged or endogenous bait proteins (Figure 3).

We will briefly explain the two most common AP approaches: (i) antibody-based immunoprecipitation of endogenous proteins and (ii) affinity tags, with critical examples related to mammalian autophagy. In antibody-based purifications, the endogenous bait along with its binding partners can be enriched in their native environment. For instance, autophagy induction by the tumor suppressor protein CDKN2A/ARF, which is a growth suppressor localizing to the nucleolus under growth conditions and to mitochondria under autophagy-inducing conditions, was discovered by an AP-MS approach. Endogenous CDKN2A and its binding partners were enriched using an anti-CDKN2A antibody prior to MS measurements. Under autophagy conditions, CDKN2A interacted with BCL2L1/Bcl-XL at mitochondria, interfering with the BCL2L1-BECN1 interaction and freeing BECN1 to bind to the PIK3C3 complex to promote autophagosome biogenesis [87]. In general, immunoprecipitations of endogenous proteins coupled to MS generate information on native interactions of baits, though there are shortcomings to this method. Firstly, antibodies can bind to different bait isoforms or splice variants, reducing the resolution of interactions. Secondly, protein interactions can be disrupted due to competition between an antibody and interacting proteins, thereby losing information on such interactions. Thirdly, testing and choosing the right high-quality antibody for the immunoprecipitation can be cost-intensive. Finally, a control antibody might not represent the same background binders even though the antibody is species and isotype-matched [88].

Years of research and tremendous improvements in protein engineering helped in developing an array of affinity tags differing in size, length, and affinity to study interactomes. These affinity tags can be classified as protein- and peptide-based tags. There are plenty of single-peptide affinity tags: HA (hemagglutinin), FLAG, MYC/c-myc, 6x/8x His, 2x SBP (streptavidin-binding peptide), and CBP (calmodulin-binding peptide), to name a few. In addition, there are protein tags e.g., GFP (green fluorescent protein), MBP (maltose binding protein), and GST (glutathione-S-transferase) [89]. Next to single tags dual tags exist, consisting of a protein domain/peptide, a cleavage site, and a second peptide tag collectively used for TAP [90]. These are widely used two-step protein purification methods to isolate rather clean protein complexes in yeast. In its initial variant, the tag consists of two IgG-binding units or Z-domains from protein A followed by a tobacco etch virus (TEV) protease cleavage site and a CBP peptide. This purification involves the usage of IgG-coupled beads, which capture the protein A domain, followed by cleavage and release of the complex by TEV protease. Using calmodulin-coupled beads, the CBP-tagged protein complex is captured and purified in a second step. Though this strategy works well in yeast cells and significantly reduces nonspecific background proteins, it was rather inefficient in mammalian cells. Another TAP tag called GS-TAP was developed to purify proteins from mammalian cells [91]. The tag consists of a protein G domain, a TEV protease cleavage site, and an SBP peptide. One advantage of using this tag is the 10-fold increased efficiency of bait purification; moreover, the two-step purification can be skipped using streptavidin beads and eluting the bait with biotin [91].

Aforementioned tags were used in many studies to uncover PPIs modulating sequential events in autophagosome biogenesis. Here, we focus on studies performed in mammalian cells and refer the reader to some excellent articles explaining in detail the studies that have so far been carried out in yeast to understand autophagy [92–95]. A list of crucial AP-MS studies that helped in uncovering the functional network of interactions modulating different events in autophagy using ATG proteins expressed in mammalian cell lines are listed in Table 1. A seminal AP-MS study on autophagy-related proteins and proteins involved in autophagosome biogenesis utilized the expression of 65 HA-tagged bait proteins, of which 32 were primary and 33 secondary baits, in HEK293T cells. Primary baits were chosen based on their functional links to autophagy and vesicle trafficking. To validate the interaction network of primary baits, secondary baits were chosen based on high interconnectivity with primary baits and functional domains or gene ontology (GO) terms linked to autophagy. Bait proteins and interaction partners were purified using anti-HA beads prior to MS measurements. The analysis of this large-scale proteomics dataset identified 2,553 potential interactors, of which 409 high-confidence candidate interaction proteins with 759 interactions were shortlisted, which revealed a global interaction network involved in mammalian autophagy. These interactions revealed the involvement of proteins with various functionalities, among others protein kinases, PtdIns3P-binding proteins, lipid transport proteins, lipid kinases, and protein ubiquitination machinery. New functional links between various proteins were revealed, as for example, association of ULK1 and ULK2 with AMPK, thus indicating a crosstalk in energy sensing. The authors performed extensive AP-MS analyses of Atg8 homologs comparing nutrient-rich and autophagy-inducing (Torin-1 treatment which inhibits MTORC1) conditions. Interestingly, the extensive overlap of interactomes from ATG8 homologs showed a functional redundancy between these proteins. This overlap of interactions is likely due to the presence of a conserved LIR docking site in ATG8 homologs interacting with cargo receptors/substrates having a conserved hydrophobic LIR [96]. GST-tagged LC3 variants were also used to identify interacting partners by LC-tandem mass spectrometry (MS/MS): e.g., GST-tagged LC3B bound to glutathione-sepharose led to the identification of FYCO1 as novel LIR-dependent LC3B interactor. FYCO1 was shown to also bind RAB7A and PtdIns3P and to promote microtubule-dependent transport of autophagic vesicles [97]. GST-tagged GABARAPL1 was employed to characterize its interaction with HSP90 family members, HSP90AA1 activity being critical for GABARAPL1 stability [98].

**Table 1.** AP-MS studies of ATG proteins

	Tagged protein (yeast/mammalian)	Affinity tag	Summary	Ref	
1.	Atg1/ULK1	Flag/HA	Mouse ULK1 interacts with RB1CC1. RB1CC1 helps in stabilizing ULK1 and mediates phagophore targeting of ULK1.		[96,146]
		Flag/S-tag	Mouse ULK1 interaction with ATG13 and RB1CC1 improves its activity and stability. Interactions between the proteins are independent. The complex localizes to the phagophore.		[147]
2.	Atg1/ULK2	HA	ULK2 interacts with RB1CC1, and ATG13 with ATG101 to form a complex. ULK2 also interacts with catalytic and regulatory subunits of AMPK.		[96]
3.	Atg2/ATG2A/B	HA	ATG2A interacts with ATG2B and WDR45.		[96]
4.	Atg3/ATG3	HA	ATG3 interacts with ATG7 and ATG12 etc.		[96]
5.	Atg4/ATG4A-D	HA	ATG4B interacts with all ATG8 homologs		[96]
6.	Atg5/ATG5	HA	ATG5 interacts with ATG12 and ATG16L1 forming a complex		[96]
		Flag	ATG5 interacts with ATG12, ATG10 and ATG16L1		[148]
7.	Vps30/Atg6/BECN1	TAP (MYC-TEV-Flag)/HA	BECN1 interacts with PIK3C3, PIK3R4, ATG14, UVRAG, and RUBCN forming three different complexes.		[96,149]
8.	Atg7/ATG7	HA	ATG7 interacts with ATG3, GABARAP, GABARAPL1 and GABARAP L2.		[96]
9.	Atg8/MAP1LC3A	HA	LC3A interacts with ATG7, FYCO1, ATG3, and SQSTM1 etc.		[96]
	Atg8/MAP1LC3B	HA	LC3B interacts with ATG7, ATG4B and ATG16L1 etc.		[96]
	Atg8/MAP1LC3C	HA	LC3C interacts with PIK3C3, ATG5, ATG3, ATG7 and ATG16L1 etc.		[96]
	Atg8/GABARAP	HA	GABARAP interacts with PI4K2, NBR1, ATG3 and ATG7 etc.		[96]
	Atg8/GABARAPL1	HA	GABARAPL1 interacts with ATG4B, ATG7, and NIPSNAP1 etc.		[96]
	Atg8/GABARAPL2	HA	GABARAPL2 interacts with WDR62, ATG7, NEDD4 and NBR1 etc.		[96]
10.	Atg9/ATG9A/B	Endogenous IP	ATG9 vesicles contain ARFIP1, ARFIP2, PI4K2A and PI4K3B which controls starvation induced autophagy.		[150]
11.	Atg10/ATG10	HA	It interacts with ATG3, ATG7, and ATG4B etc.		[96]
12.	Atg11/Atg17/RB1CC1	HA	RB1CC1 interacts with ULK1, ATG101 and ATG13.		[96]
13.	Atg12/ATG12	HA	It interacts with ATG3, ATG5, ATG16L1 and ATG7.		[96]
14.	Atg13/ATG13	Flag	ATG13 interacts with ULK1 and RB1CC1 to form a trimeric complex and maintains stability of ULK1.		[151]
		HA	ATG13 interacts with ATG101 and RB1CC1.		[96]
		Flag	ATG101 interacts with ATG13 to form the tetrameric complex with ULK1 and RB1CC1.		[152]
15.	Atg14/ATG14	HA	It interacts with PIK3C3, BECN1, and PIK3R4 forming a PtdIns3K-I complex.		[96]
16.	Atg16/ATG16L1	HA	It interacts with ATG5 and ATG12 to contribute to the ATG8 conjugation pathway.		[96]
17.	Atg18/WIPI1	GFP	It interacts with WIPI2B supporting its PtdIns3P effector function.		[24]
	Atg18/WIPI1	HA	It interacts with ATG2A and WIPI2.		[96]
	Atg18/WIPI2B	GFP	It interacts with ATG12–ATG5-ATG16L1, WIPI1 and co-associates with WDR45/WIPI4.		[24]
	Atg18/WIPI2B	GFP	It interacts with ATG16L1 and ATG5.		[29]
	Atg18/WIPI2	HA	It interacts with ATG2A, BTBD8 and NUDC.		[96]
	Atg18/WIPI2	HA-Flag	WIPI2 interacts with ATG5.		[153]
	Atg18/WDR45B	GFP	It interacts with TSC2 complex acting as a scaffold for RB1CC1.		[24]
	Atg18/WDR45	GFP	It interacts with ATG2A/2B to enhance autophagosome membrane formation.		[24]
18	Atg29, Atg31/ATG101	Flag	ULK1/2 (mouse) and RB1CC1 interact with ATG101. ATG101 interacts only with ATG13 to form the tetrameric complex with ULK1 and RB1CC1		[152]
		HA	ATG101 interacts with ATG13		[96]

Moreover, AP-MS approaches were used to analyze organellar compositions, representing a valuable alternative approach for the characterization of cellular stress-response pathways. SILAC-labeled MCF-7 cells expressing eGFP-LC3 were utilized to compare autophagosome proteome changes upon different stress conditions. Vesicle fractionations in combination with anti-GFP-based enrichments, were coupled to MS analyses to reveal stimulus-dependent autophagosome proteomes [99]. Affinity-purified lysosomes were used to profile proteome changes under nutrient-rich and autophagy conditions. The authors expressed 3x HA-tagged TMEM192, a lysosomal membrane protein, in HEK293T cells and performed anti-HA AP to enrich lysosomes prior MS-based identification and quantification. They identified a change in the localization of a protein called NUFIP1 (nuclear FMR1 interacting protein 1), which shifted from the nucleus toward lysosomes/autophagosomes during starvation-induced autophagy and suggested that NUFIP1 might act as a potential receptor for ribophagy [100]. However, in a recent unbiased approach that studied the contributions of protein translation and degradation to ribosomal protein abundance, the role of NUFIP1 in ribosomal protein degradation could not be confirmed, questioning its function in ribophagy [101].

Post-translational modifications such as phosphorylation, ubiquitination, and sumoylation modulate interactions between autophagy proteins at a particular spatial and temporal resolution [102]. For example, AP-MS studies were performed to identify phosphorylation sites on ULK1. N-terminal TAP-tagged mouse ULK1 was expressed and purified from HEK293T cells prior to LC-MS/MS measurements. A total of 16 novel phosphorylation sites on ULK1 were identified, which also included autophosphorylation sites. The authors suggested that phosphorylation at Ser867 and Ser913 of ULK1 might promote its association with ATG13 and RB1CC1 to form an active autophagy initiation complex [103].

AP methods coupled to either western blot or MS allow analyses of protein complexes in all organelles and compartments without the requirement of prefractionation. The aforementioned examples highlight the robustness of these approaches in identifying PPIs and PPI-modulating PTMs to shed light on the molecular mechanisms in autophagy pathways. However, these approaches also have shortcomings. In general, co-purifying contaminants or nonspecific proteins, either binding to the solid matrix or the antibody, are also enriched, making it sometimes hard to distinguish false and true interactors [104,105]. Usage of quantitative proteomic approaches and analyzing data against contaminant repositories, such as Contaminant Repository for Affinity Purification MS Data (CRAPome), can help in identifying contaminants [106]. Due to overexpression, bait proteins are prone to problems like altered localization, protein misfolding, and aggregation. However, bait levels can be controlled via inducible promoters. In addition, CRISPR approaches are now being used to perform genome editing, enabling the expression of tagged proteins at endogenous levels [107]. Usage of either CRISPR knockout or RNAi approaches that remove the endogenous protein can be efficiently used as negative controls [86,88,108]. Also, the localization of the affinity tag, either at the N- or C-terminus of the bait, has to be critically evaluated. In rare cases, internal tags have been used to address specific biochemical questions such as linear ubiquitination [109]. For the analysis of endogenous proteins, choosing the right mammalian cell line is essential. Commonly, cells expressing high amounts of bait proteins simplify subsequent mechanistic studies.

Critically, AP-MS data are commonly binary and do, therefore, neither yield insights into the structure (and stoichiometry) of complexes nor differentiate between direct and indirect interaction partners. Importantly, AP can isolate only stable or strong bait interactors with nanomolar or higher affinities; thus, dynamic, weak, and transient interactions are often missed [110].

### Proximity labeling-mass spectrometry to study weak protein-protein interactions and protein neighborhoods

**Figure 4.**
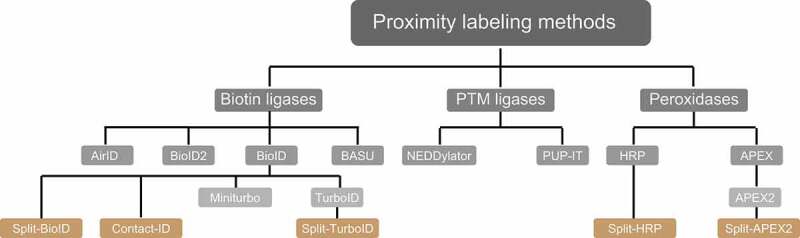
Proximity labeling enzymes to study protein neighborhoods. Proximity labeling ligases can be classified into three types based on their activity: biotin ligases, PTM ligases and peroxidases. Biotin ligases (BioID, BioID2, AirID and BASU) catalyze the conversion of biotin to a reactive biotin intermediate, which labels lysine residues of proximal proteins. In presence of H_2_O_2_, peroxidases (APEX2 and HRP) convert biotin phenol to biotin-phenoxyl radical, which labels electron-rich amino acid residues such as Tyr. PTM ligases add peptide/protein tags to proximal proteins

**Figure 5.**
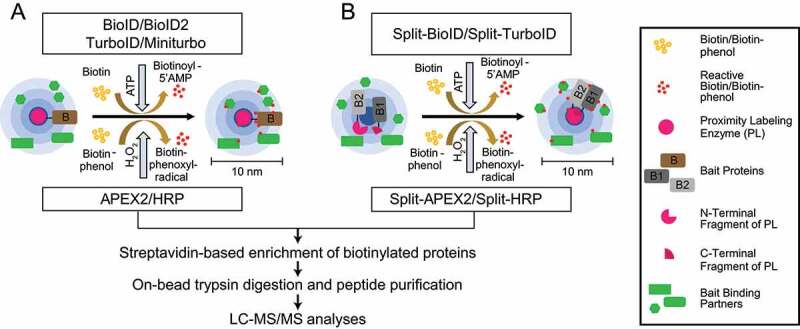
PL-MS approaches. (A) Biotin ligases and peroxidases generate reactive biotin/biotin-phenol intermediates, respectively, which tag proximal proteins. (B) Split variants of biotin ligases and peroxidases are used to identify proximal proteins, while two bait proteins interact to form a complex. Interactions of proteins bring the N/C terminal fragments of the ligases in close proximity allowing the formation of an active PL holo-enzyme. After cell lysis, biotinylated proteins are enriched using classical streptavidin-based enrichment prior to bottom-up LC-MS/MS analysis

PL-MS-based quantitative proteomics enables the identification and characterization of weak transient PPIs and neighborhoods. To this end, different classes of enzymes, such as biotin ligases, PTM ligases, and peroxidases, are used to label protein neighborhoods (Figure 4) [111–113]. These enzymes are fused to bait proteins and catalyze the covalent transfer of a chemical group to proximal proteins. Biotin ligases are enzymes that catalyze, in an ATP-dependent reaction, the conversion of biotin to a reactive biotinoyl-5ʹAMP intermediate. Biotinoyl-5ʹAMP reacts with exposed lysine residues of proximal proteins, leading to the covalent attachment of biotin within an estimated radius of 10 nm around the enzyme [114,115]. A classical proximity-labeling technique, called proximity-dependent biotin identification (BioID), utilizes the biotin ligase BirA, a monomeric 35.4-kDa protein, from *Escherichia coli* that has been further engineered to BirA^R118G^ to improve its catalytic activity (also referred to as BirA*, with “*” indicating its promiscuous activity). Cells expressing BioID-fused bait proteins are incubated with biotin for 16–24 h to reach maximum labeling efficiency. This relatively long incubation time is required to generate sufficient proximally biotinylated proteins, which can then be subsequently enriched using classical high-affinity streptavidin or neutravidin beads prior to LC-MS/MS sample preparation and measurements (Figure 5A) [116].

Introducing further mutations to BioID improved its labeling efficiency several-fold and led to two other commonly used biotin ligases called TurboID and miniTurbo [117]. Compared to wild-type BirA, the improved 35 kDa TurboID variant contains a total of 15 mutations, amongst these a substitution of Arg118 to Ser. By deleting the N-terminal DNA-binding domain, a slightly smaller version of TurboID of 28 kDa, called miniTurbo, has also been generated. MiniTurbo harbors 13 out of the 15 mutations of TurboID. These two tags help in identifying snapshots of protein interactions using labeling times of only 5–15 min (Table 2). A drawback of TurboID is its high affinity toward biotin, which can lead to the usage of endogenous biotin, thereby disrupting biotin-dependent metabolic pathways and leading to toxicity in certain model organisms [117]. A smaller version of BirA of 28 kDa, BioID2, which naturally lacks the N-terminal DNA-binding domain, was identified in the thermophile *Aquifex aeolicus*. The interesting feature of this enzyme is the reduced background of DNA/chromatin interacting proteins due to absence of the DNA-binding domain [118]. Overall, labeling time and activity of BioID2 are comparable to the one of BioID. Another biotin ligase variant, termed BirA from *Bacillus subtilis* (BASU), was engineered without DNA-binding domain. This ligase was used to identify novel RNA-binding and proximal proteins; the respective approach was termed RNA-protein interaction detection (RaPID) [119]. For this, BASU was fused to the λN peptide, which binds bacteriophage lambda BoxB stem-loops. BoxB stem-loops are synthesized to flank the respective RNA-of-interest. Binding of the ligase to the stem-loop structure led to biotinylation of proteins binding to the specific RNA-of-interest within a 10 nm radius. At labeling times of 1 min, BASU showed more than >30-fold increased signal-to-noise ratios compared to BioID and BioID2 [119]. One of the most recent technical developments is the generation of ancestral BirA for proximity-dependent biotin identification (AirID). Metagenome data and ancestral sequence reconstruction coupled to site-directed mutagenesis gave rise to this BirA variant with 82% sequence similarity to BioID, which labels proteins *in vitro* or *in vivo* in 3 h using lower concentrations of biotin than in the original approach [120].

**Table 2.** Commonly used PL methods

Method	Mutations	Substrate	Labeling time	Labeling residue	Usage	Ref
BioID	R118G	Biotin	16–24 h	Lys	Classical enzyme used to identify PPIs; requires long labeling time.	[116]
Split-BioID	(i) E140/Q141 or (ii) E256/G257	Biotin	16–24 h	Lys	It is used as a PCA method to identify proximal proteins of binary interactions. Activity is lower than of BioID.	[130,131]
BioID2	R40G	Biotin	16–24 h	Lys	A natural smaller version of 28 kDa having higher affinity toward biotin compared to BioID.	[118]
TurboID	Q65P, I87V, R118S, E140K, Q141R, A146Δ, S150G, L151P, V160A, T192A, K194I, M209V, M241T, S263P, I305V	Biotin	10 min	Lys	A 35-kDa protein with improved activity compared to its parental enzyme BioID. Mostly used to capture snapshots of PPIs.	[117]
Split-TurboID	L73/G74	Biotin	4 h	Lys	It is used as a PCA method to identify proximal proteins of organelle contact sites. Activity is higher than of split-BioID.	[154]
Contact-ID	G78/G79	Biotin	16 h	Lys	Identifying proteins at membrane contact sites.	[155]
AirID	R118S, G26, F124, V171 and A297	Biotin	3 h	Lys	A faster PL method compared to classical BioID, Contact-ID and BioID2.	[120]
miniTurbo	Residues 1–63 deleted and Q65P, I87V, R118S, E140K, Q141R, A146Δ, S150G, L151P, V160A, T192A, K194I, M209V, I305V	Biotin	10 min	Lys	Slightly smaller version of 28 kDa compared to TurboID having the potential to reveal snapshots of PPIs.	[117]
BASU	R124G, E323S, G325R	Biotin	1 min	Lys	More active version than BioID used to identify RNA binding proteins	[119]
APEX2	K14D, W41F, E112K, A134P	Biotin-phenol	1 min	Tyr (Trp, Cys, Phe)	It is used as an EM tag. It gives a snapshot of PPIs due to fast labeling time.	[126]
Split-APEX2	K22R, R24G, G50R, K61R, H62Y, N72S, P125L, I165L, I185V	Biotin-phenol	1 min	Tyr (Trp, Cys, Phe)	It is used as a PCA method to identify proximal proteins of binary interactions.	[134]
HRP	-	Biotin-phenol	1 min	Tyr (Trp, Cys, Phe)	Inactive in cytosol. It is majorly used to study cell surface proteins and secretory pathways. It is also used as EM tag.	[127]
Split-HRP	Split at aa 213; T21I, P78S, R93G, N175S, N255D, L299R	Biotin-phenol	1 min	Tyr (Trp, Cys, Phe)	Inactive in cytosol. It is a PCA assay used to identify PPIs involved in cell-cell communication.	[132]
PUP-IT	-	Pup	-	Lys	Specifically designed to study membrane PPIs. Labeling time depends on expression levels of the bait.	[121]
NEDDylator	-	NEDD8	18 h	Lys	Identification of proteins binding to small molecules and proteins.	[122,123]

PTM ligases are enzymes that commonly add tags onto lysine residues of bait-proximal proteins. Pupylation-based interaction tagging (PUP-IT) is a method that was developed to identify PPIs at membranes. This approach involves the expression of a bait protein fused to the bacterial Pup ligase PafA and co-expression of a Pup (prokaryotic ubiquitin-like protein) variant with a C-terminal Gly-Gly-Glu sequence. The Pup ligase catalyzes the phosphorylation of the C-terminal Glu residue, leading to its activation and conjugation to lysine residues of proteins that are in proximity to the bait. Due to the low diffusible nature of the activated Pup tag a smaller labeling radius is achieved, thus reducing background. The major shortcoming of this method is the large size of ~54 kDa of the ligase and its low catalytic activity, which render it inadequate to capture snapshots of PPIs [121]. Neddylation by the 76 amino acids large protein NEDD8 is a ubiquitin-like modification, which has been exploited to identify PPIs. For this, a modified version of the NEDD8 E2-conjugating enzyme UBE2M/UBC12 called NEDDylator is fused to a protein or a small molecule. The specific tagging of NEDD8 to proximal proteins happens via the nucleophilic attack of prey lysine epsilon amino groups to thioester-bound NEDD8 on the bait-NEDDylator. This confirms the direct contact between bait and prey proteins, which ensures neddylation of potential binding partners. This tool has been successfully coupled to SILAC-based quantitative MS to identify small molecule-protein and protein-protein interactions in mammalian cells [122,123].

Peroxidases are oxidoreductases that catalyze redox reactions in the presence of hydrogen peroxide. This includes APEX (ascorbate peroxidase) and HRP (horseradish peroxidase) enzymes, which catalyze the conversion of biotin-phenol to biotin-phenoxyl radical in the presence of hydrogen peroxide (H_2_O_2_) (Figure 5A). This membrane-impermeable reactive radical covalently labels electron-rich amino acids, majorly Tyr and to some extent Trp, Cys, and Phe, of proteins proximal to the bait within a 10 nm radius. APEX was initially identified in pea and engineered to reduce its dimerization property and improve its catalytic activity. This enzyme contributes to polymerization and deposition of diaminobenzidine for electron microscopy (EM) studies. APEX2 from soybean, a 28-kDa enzyme, was engineered to improve its catalytic activity allowing labeling times of 1 min or less in mammalian cells [124–126]. HRP is another peroxidase of 44 kDa used for both EM and PL studies [127]. Due to its inactivity in cytosol, it is majorly used to study cell surface molecules and secretory pathways targeting the enzyme via either ligands or antibodies to cell surfaces, using techniques like selective proteomic proximity labeling assay using tyramide (SPPLAT) and enzyme-mediated activation of radical sources (EMARS) [128]. Alternatively, antibody-conjugated HRP was used intracellularly in fixed tissues and cells to perform PL of bait proteins in techniques such as biotinylation by antibody recognition (BAR) [129]. Overall, due to its usage in EM studies and its fast labeling time, APEX2 is the most widely used technique revealing snapshots of PPIs and enabling analyses of stimulus-dependent interactomes. However, APEX2 is less active compared to HRP and sensitive to H_2_O_2_-mediated inhibition.

Notably, tremendous improvements have been made in protein fragment complementation assays (PCA) coupled to PL approaches. Split variants of labeling enzymes were generated using inactive N- and C-terminal fragments of BioID or APEX2 (Figure 5B). The respective fragments are fused to two different baits known to interact with each other. Interaction between the baits enables the reconstitution of the holo-enzyme allowing biotinylation of proximal proteins. This technique enables identification of interactions depending on interaction of two bait proteins at a specific spatial and temporal resolution. Split-BioID was the first variant of this approach [130]; however, longer labeling time and lower activity of the reconstituted complex made this split version non-suitable to study dynamic interactions [131]. These hurdles were resolved with the recent discovery of split versions based on HRP, APEX2, and TurboID. Split HRP was used, for example, to study cell-cell interactions [132]. The initially made split-APEX2 was less active compared to its full-length variant and led to a second split version with additional nine mutations, which improved activity and specificity [133,134]. Also recently, a version of split-TurboID was developed for the analyses of PPI of complexes, organelle and cell contact sites [135]. For more technical information, we would like to refer readers to recent reviews [111–113]. In the remainder of this manuscript, we focus on studies using PL approaches coupled to MS to understand autophagy mechanisms.

Up to date, only a few studies utilized PL coupled to MS-based quantitative proteomics to uncover the roles of proteins involved in autophagy (Table 3). TBC1D14, a TBC domain containing protein, has a strong effect on the structure and function of recycling endosomes and negatively regulates the formation of autophagosome upon overexpression [136]. BioID coupled to MS analysis of TBC1D14 identified its interaction with TRAPPC8, a subunit of trafficking protein particle III (TRAPP-III), a multimeric protein complex with guanine exchange factor (GEF)-activity toward RAB1B, promoting its GTP-loaded state. TRAPP-III regulates the cycling of ATG9 from early endosomes and the Golgi apparatus to the ATG9 compartment. Overexpression of TBC1D14 led to mislocalization of TRAPPC8 onto recycling endosome tubules, to a fragmented Golgi apparatus, and to disruption of the Golgi ATG9 pool leading to inhibition of autophagosome formation [137].

**Table 3.** PL-MS studies in autophagy research

	Bait	Method	Quantitative MS method	Summary	Ref
1.	AP4E1/M1	MYC-BioID	Label-free	Identification of AP-4 subunits, TEPSIN, ATG9, AP-4 complex accessory proteins-RUSC1 and RUSC2. Role of AP-4 in ATG9 trafficking and autophagosome biogenesis.	[156]
2.	LGALS8	APEX2	Label free	Role of LGALS8 in MTORC1 inactivation during lysosomal damage via Regulator-RRAG signaling.	[141]
3.	LGALS3	APEX2	SILAC	LGALS3 helps in recruiting ESCRT complex and PDCD6IP to promote repair of damaged lysosomal membranes. LGALS3 also promotes autophagy of lysosomes via its interactions with TRIM16.	[144]
4.	LGALS9	APEX2	SILAC	Lysosomal damage is sensed by LGALS9, and along with ubiquitin, it signals binding of autophagy receptors to promote lysosome degradation. LGALS9 and ubiquitin cooperatively activates AMPK for autophagy induction.	[142]
5.	MAP1LC3A	MYC-APEX2	SILAC	Identification of 779 neighboring proteins.	[107]
	MAP1LC3B	MYC-APEX2	SILAC	Identification of 622 neighboring proteins out of which some were upregulated by BafA1: SQSTM1, NBR1, and PCM1 etc.	[107]
	MAP1LC3C	MYC-APEX2	SILAC	Identification of 762 neighboring proteins out of which some were upregulated by BafA1: PAICS, SQSTM1, and MTX1, etc.	[107]
	GABARAP	MYC-APEX2	SILAC	Identification of 537 neighboring proteins out of which some were upregulated by BafA1: SQSTM1, etc.	[107]
	GABARAPL1	MYC-APEX2	SILAC	Identification of 405 neighboring proteins out of which some were upregulated by BafA1: IMPDH2, PAICS, and HSP90AA1 etc.	[107]
	GABARAPL2	MYC-APEX2	SILAC	Identification of 494 neighboring proteins out of which some were upregulated by BafA1: SQSTM1, PAICS, and ATP6V0D1, etc.	[107]
	MAP1LC3B	MYC-BioID	SILAC	Large overlap of APEX2 and BioID-tagged LC3B proximal proteomes.	[107]
	MTX	MYC-APEX2	SILAC	Substantial overlap between MTX1 and LC3C proximal proteome.	[107]
6.	OPTN-TAX1BP1	APEX2	TMT	Identification of essential factors involved in the formation of mitochondria-autophagosome synapse and for selective degradation of mitochondria.	[140]
7.	STK38	APEX2	SILAC	STK38 a Ser/Thr kinase, phosphorylate XPO1 (exportin) to mediate its export from the nucleus along with BECN1and YAP1. Cytosolic localization of XPO1 is crucial in starvation-induced autophagy.	[157]
8.	TBC1D14	MYC-BioID	Label free	TBC1D14 interacts and traps one of the subunits of TRAPP, TRAPPC8 which inhibits starvation induced autophagosome formation.	[137]
9.	TEX264	APEX2	TMT	TEX264 interacts with autophagy receptors: SQSTM1, CALCOCO2 and TAX1BP1, ER membrane proteins: CANX, CISD2, and autophagy regulators: ATG14, and WIPI2 during starvation. TEX264 get degrades in a LIR-dependent manner showing its role as a potential receptor in reticulophagy.	[158]

APEX2 labeling of human ATG8 homologs coupled to MS-based quantitative proteomics was used to analyze autophagosome content. APEX2-LC3C labeling identified a reproducible interaction of LC3C with a protein called MTX1 (metaxin 1). MTX1 binds to SAMM50 located on the outer mitochondrial membrane. Together with MTX2 bound to the cytosolic face of MTX1, these proteins form the sorting and assembly machinery (SAM) complex. This complex, together with the mitochondrial contact site and cristae junction organizing system (MICOS), maintain cristae structure, mitochondrial morphology, and homeostasis. Colocalization studies and functional biochemical analyses of MTX1 revealed its role in autophagic clearance of parts of damaged mitochondria in a piecemeal fashion via LC3C and SQSTM1 [107]. Recently, new roles of LC3s in protein secretion were identified using PL-MS. RNA-binding proteins and small non-coding RNAs were shown to be packed into extracellular vesicles in a MAP1LC3B and LC3-conjugation-machinery-dependent manner [138]. Also, GABARAP was identified in extracellular vesicles using cells expressing APEX2-GABARAP. Interestingly, these extracellular vesicles also contained proteins with which GABARAP was shown to interact inside autophagosomes, further supporting a crosstalk between autophagy and protein secretion [139].

APEX2 labeling of the mitophagy receptors OPTN, OPTN^D474N^ a ubiquitin-binding defective mutant, and TAX1BP1 in HeLa cells coupled to TMT (8/9 plex)-based quantitative MS analysis was performed comparing antimycin A/oligomycin A (inducing depolarization of mitochondria) treatments for 1 and 3 h with non-treated cells to identify proteins proximal to the receptors during mitophagy. In combination with a CRISPR-based genetic screen coupled to mitophagy flux assays, HK2 (hexokinase-2) was characterized as a scaffold, forming a 700-kDa complex consisting of PINK1-PRKN and other ubiquitinated proteins, essential for the clearance of damaged mitochondria [140].

The role of galectins in maintenance, repair, removal, and biogenesis of lysosomes upon injury has been extensively characterized using APEX2 labeling coupled to quantitative MS. Galectins are beta-galactoside binding proteins with an intrinsic carbohydrate recognition domain (CRD). This property helps in sensing membrane damage due to exposure of membrane glycans. LGALS8 (galectin 8) was shown to induce autophagy upon endomembrane damage by regulating MTORC1 activity via changes in the activation state of RRAG GTPases [141]. APEX2-LGALS9 labeling revealed its role in lysosomal damage sensing via activation of AMPK. Upon lysosomal damage, LGALS9 displaces the deubiquitinase USP9X from MAP3K7/TAK1 kinase, thereby promoting K63-linked polyubiquitination and activation of the kinase. MAP3K7 in turn activates AMPK and thereby autophagy by phosphorylation of Thr172 [142]. Previously, LGALS3 was known to promote TRIM16 based autophagic removal of damaged lysosomes and activate TFEB, a transcription factor for lysosome biogenesis [143]. In addition, APEX2-LGALS3 labeling was performed to understand its role in the repair and clearance of endomembrane damage via autophagy. LGALS3 helped in recruiting the ESCRT component PDCD6IP/ALIX upon lysosomal membrane damage. LGALS3 promoted the interaction of PDCD6IP with CHMP4B, which collectively mediated scission and closure of lysosomal membranes [144].

Thus, the aforementioned examples show the impact of PL-based MS in understanding autophagy-relevant mechanisms. PL provides information about the proximal proteome change around the bait of interest at a given spatial and temporal resolution. However, PL methods reveal many proximal neighbors and potentially interacting proteins leading to an inherently high background. Therefore, the controls used to distinguish true versus false-positive interactors are critical. Commonly, five different types of controls are employed: (i) cells without any PL enzyme treated with biotin/biotin-phenol, (ii) the enzyme fused to an unrelated protein such as GFP or RFP, (iii) the enzyme fused to an inactive or mutant version of the respective bait, (iv) the enzyme fused to a compartment-specific, unrelated protein, which localizes similarly to the bait, and (v) cells with the free PL enzyme [111,145]. Given the variety of controls with their intrinsic pros and cons, choosing the right control for the desired PL experiment is still a debate in the field. We see large differences in enrichments of baits and their binding partners depending on the used control conditions (unpublished data). Whereas control (i) leads to high enrichment rates, the number of false positives appears high. In contrast, due to the high activity of free enzymes in control (v), enrichment rates are low, and the number of false-negatives appears high, i.e., weak transient interactions seem to be lost. Thus, we favor controls (ii)-(iv) in which PL enzyme-tagged bait proteins are compared to PL enzyme-tagged control/unrelated proteins. By using inducible expression systems, PL enzyme-bait protein amounts can be titrated to avoid high expression levels, which again increases background signals of nonspecifically enriched proteins. Additionally, one should consider that BioID-based approaches rely on the availability of accessible lysine residues and APEX2 methods on electron-rich amino acid residues, i.e., tyrosine, in proximal proteins. Importantly, problems could arise from toxicity issues related to overexpressed PL enzymes e.g., via protein aggregation, mislocalization, and functional inactivation of baits due to the fused PL enzyme. Hence, experimental characterizations of PL enzymes and controls are essential to design meaningful experiments for studying complex biological questions.

## Conclusions and outlook

Over two decades, there has been a significant increase in the understanding of mechanisms regulating autophagy. Technological improvements contributed to the expanding list of autophagy-related and -associated proteins. MS-based proteomics studies helped tremendously in identifying and characterizing relevant proteins. Overall, various factors influence the identification of PPIs, like the range of affinities and composition of complexes, next to intrinsic methodological shortcomings of the used analytical approach. Thus, in-depth knowledge of potential methods and understanding of influential experimental factors that might modulate PPIs is essential for choosing appropriate approaches to characterize any PPI. Here, we introduced various MS-based methods available to study PPIs, explained in detail principles and applications of AP-MS- and PL-MS-based approaches, and listed examples of autophagy relevant studies. There are still various unanswered, mechanistic questions like: which membrane sources are employed under which conditions for autophagosome biogenesis? Which factors influence the localization of autophagy initiation complexes at ER sites? Which triggering factors regulate the balance between selective and nonselective autophagy, and how do proteins modulate autophagosome size and shape under these conditions? Extensive characterizations of PTMs modulating PPIs are essential for understanding the above questions. In the future, it will be essential to combine the analyses of PTMs and PPIs. The increasing scanning speed and sensitivity of mass spectrometers will help in generating more detailed views of the regulation of PTMs. Both DIA and DDA methods will likely contribute to generating truly comprehensive datasets that will also be useful for systems biology-based approaches. However, the limited dynamic range of mass spectrometers still poses a challenge to be addressed. Modified peptides still have to be enriched, but enrichment approaches often differ depending on which PTMs are analyzed, impeding the study of PTM crosstalk. Top-down proteomics approaches might partially address this issue. Finally, the growing field of MS-based lipidomics will be essential to fully understand the membrane dynamics underlying autophagy. Thus, we believe that cutting-edge MS approaches will continue to help to address autophagy-related questions and lead to a comprehensive understanding of autophagy-related processes.
